# Nuclear genomic control of naturally occurring variation in mitochondrial function in *Drosophila melanogaster*

**DOI:** 10.1186/1471-2164-13-659

**Published:** 2012-11-22

**Authors:** Patricia Jumbo-Lucioni, Su Bu, Susan T Harbison, Juanita C Slaughter, Trudy FC Mackay, Douglas R Moellering, Maria De Luca

**Affiliations:** 1 Department of Nutrition Sciences, University of Alabama at Birmingham, Birmingham, AL, 35294, USA; 2 Laboratory of Systems Genetics, National Heart Lung and Blood Institute, Bethesda, MD, 20892-1654, USA; 3 Department of Genetics, North Carolina State University, Raleigh, NC, 27695, USA; 4 Present address: Department of Biological Sciences, Vanderbilt University, Nashville, TN, 37232, USA

## Abstract

**Background:**

Mitochondria are organelles found in nearly all eukaryotic cells that play a crucial role in cellular survival and function. Mitochondrial function is under the control of nuclear and mitochondrial genomes. While the latter has been the focus of most genetic research, we remain largely ignorant about the nuclear-encoded genomic control of inter-individual variability in mitochondrial function. Here, we used *Drosophila melanogaster* as our model organism to address this question.

**Results:**

We quantified mitochondrial state 3 and state 4 respiration rates and P:O ratio in mitochondria isolated from the thoraces of 40 sequenced inbred lines of the Drosophila Genetic Reference Panel. We found significant within-population genetic variability for all mitochondrial traits. Hence, we performed genome-wide association mapping and identified 141 single nucleotide polymorphisms (SNPs) associated with differences in mitochondrial respiration and efficiency (*P* ≤1 × 10^-5^). Gene-centered regression models showed that 2–3 SNPs can explain 31, 13, and 18% of the phenotypic variation in state 3, state 4, and P:O ratio, respectively. Most of the genes tagged by the SNPs are involved in organ development, second messenger-mediated signaling pathways, and cytoskeleton remodeling. One of these genes, *sallimus* (*sls*), encodes a component of the muscle sarcomere. We confirmed the direct effect of *sls* on mitochondrial respiration using two viable mutants and their coisogenic wild-type strain. Furthermore, correlation network analysis revealed that *sls* functions as a transcriptional hub in a co-regulated module associated with mitochondrial respiration and is connected to *CG7834*, which is predicted to encode a protein with mitochondrial electron transfer flavoprotein activity. This latter finding was also verified in the *sls* mutants.

**Conclusions:**

Our results provide novel insights into the genetic factors regulating natural variation in mitochondrial function in *D. melanogaster*. The integrative genomic approach used in our study allowed us to identify *sls* as a novel hub gene responsible for the regulation of mitochondrial respiration in muscle sarcomere and to provide evidence that *sls* might act via the electron transfer flavoprotein/ubiquinone oxidoreductase complex.

## Background

Mitochondria are organelles found in nearly all eukaryotic cells that participate in many fundamental cellular processes. A primary role of mitochondria is to utilize oxygen and nutrients to form adenosine triphosphate (ATP) via a process called oxidative phosphorylation (OxPhos) [1]. In addition, mitochondria are important in cellular Ca^2+^ signaling, the regulation of apoptosis, and as a main source of reactive oxygen species (ROS) [2]. ROS are generated and coordinated by redox-coupled reactions in multiple sites within the mitochondrial electron transport chain (ETC) and play critical roles in retrograde signaling [3] and physiological cell signaling and transduction [4]. However, if produced in excess, ROS can oxidize and damage various cellular components, including mitochondrial proteins, membranes, lipids, and nuclear and mitochondrial genomes [5]. Thus, mitochondrial dysfunction and ROS formation can have widespread adverse effects on many cellular processes and have been implicated in pathological conditions as diverse as heart failure, hypoxia, diabetes, neurodegenerative diseases, and the physiological process of aging [5].

The OxPhos system consists of five large multi-protein complexes, four of which (complexes I-IV) make up the ETC [1]. During OxPhos, electrons from reduced substrates, such as nicotinamide adenine dinucleotide (NADH) and flavin adenine dinucleotide (FADH_2_), which are generated in the Krebs cycle, are fuelled into complexes I (NADH dehydrogenase) and II (succinate dehydrogenase) of the ETC. The electrons are then transferred through the complexes III (cytochrome *bc*_1_ oxidoreductase) and IV (cytochrome *c* oxidase) ultimately reducing oxygen to water, with protons concurrently pumped across the mitochondrial inner membrane in complexes I, III, and IV. This establishes an electrochemical potential difference across the inner membrane and a motive force for protons to re-enter through ATP synthase (complex V). ATP synthase captures the potential energy released upon protons re-entry by converting adenosine diphosphate (ADP) and inorganic phosphate to ATP. In this manner, electron transport is coupled to OxPhos [1]. The efficiency with which mitochondria convert oxygen into ATP to perform useful work is known as mitochondrial energy coupling efficiency or P:O ratio [6]. In a perfectly coupled system, protons would only re-enter the mitochondrial matrix through ATPsynthase in the presence of ADP. In isolated mitochondrial suspensions, this form of respiration is classified as ‘state 3’ (i.e. the O_2_ is consumed in the presence of saturating amounts of respiratory substrate and ADP). However, it has been known for several decades that under normal conditions protons leak back through the mitochondrial membrane into the matrix via a mechanism that does not involve ATP synthase [7]. This *uncouples* respiration from OxPhos. Proton leak increases exponentially with the membrane potential (“non-Ohmic” pattern) [8] and is greatest under non-phosphorylating conditions, such as ‘state 4’ respiration (i.e. O_2_ is consumed in the presence of respiratory substrate and absence of ADP) in isolated mitochondria. Thus, mitochondria in the intact cell would normally respire at a rate somewhere between state 3 and state 4 respiration rates depending on the energy demand, substrate availability, oxygen, ADP availability, and proton leak back into the matrix. The mechanisms that account for proton leak are poorly understood, but phospholipids and fatty acid composition of the mitochondrial inner membrane and the expression and activation of uncoupling proteins (UCPs) are proposed contributors to the leak [9,10].

In all Metazoa, the large OxPhos complexes are encoded mainly by nuclear genes and, to a small extent, by mitochondrial genes. The only exception is represented by complex II subunits that are entirely encoded by nuclear genes [11]. Given the impact mitochondria have for cellular survival and function, numerous mutations in both mitochondrial- and nuclear-encoded OxPhos genes have been reported to be responsible for rare pathological disorders [12]. Genetic studies have also provided evidence of associations between mitochondrial DNA (mtDNA) polymorphisms and aging [13] as well as age-related metabolic disorders, such as type-2 diabetes and cardiovascular disease [14]. However, despite substantial progress in the field, we remain largely ignorant about the genomic regulation of natural variation in mitochondrial respiration. This is important for understanding the evolution of these traits in natural populations and also essential for the development of mitochondria-specific therapeutic strategies for the treatment and prevention of disorders related to mitochondrial dysfunction. To address this critical gap in our knowledge of mitochondrial biology, in this study we used *Drosophila melanogaster* as our model organism. We chose *D. melanogaster* for several reasons. First, this organism has emerged in recent years as a powerful model to elucidate the genomic basis that controls naturally occurring variation in quantitative traits, such as mitochondrial respiration traits [15]. Second, the OxPhos system of insect mitochondria resembles that of mammalian mitochondria [16-18], with the mitochondrial respiration being affected by the same inhibitors and uncouplers that affect the mammalian system [19,20]. Third, *D. melanogaster* possesses four genes coding for close relatives of the UCPs. One of the four fly genes (*Bmcp*) has been shown to be a Drosophila mitochondrial uncoupler of OxPhos [21]. Finally, several genetic mechanisms controlling energy metabolism and homeostasis are shared between invertebrates and mammals [22-25]. Thus, insights gained from genomic studies in Drosophila are likely to apply to mammals.

First, we investigated whether there is variability in mitochondrial respiration and coupling efficiency among 40 inbred, sequenced lines of the Drosophila Genetic Reference Panel (DGRP), a newly established *D. melanogaster* genomics resource [26]. This was accomplished by quantifying state 3 and state 4 respiration rates and the P:O ratios in mitochondria isolated from the thoraces (mainly composed of flight muscles) of young flies using NADH-linked respiratory substrates. Our study revealed significant genetic variation in mitochondrial function. As such, we next sought to identify the genomic architecture underlying such variability. Mitochondrial OxPhos is under dual genetic control, therefore genetic variation in both mitochondrial and nuclear genes and/or genetic interactions between nuclear and mitochondrial alleles (intergenomic epistasis) could be responsible for the observed variability in mitochondrial function [27]. Previous studies by Ballard and co-workers have provided empirical evidence that variation in the mitochondrial genome influence multiple aspects of respiration in wild-caught *D. simulans* flies [28,29]. However, much less is known about the effects of variation in nuclear genes on mitochondrial respiration, despite the fact that mostly nuclear genes are involved in mitochondrial metabolism. For example, while both nuclear and mitochondrial genes encode the respiration subunits, their transcription rely on nuclear-encoded factors. Some of these factors are directed to the mitochondria, where they control the transcription of mitochondrial DNA (mtDNA). Others act on nuclear genes required for the assembly and function of the respiratory chain [30]. Also, it is well established that to adjust the rate of ATP production to both short term and long term changes in cellular energy demand, mitochondrial respiration is subject to complex regulation via reversible phosphorylation of OxPhos enzyme complexes [31]. Additionally, a growing body of evidence points to a critical role for second messenger-mediated signal transduction mechanisms in the regulation of mitochondrial OxPhos [32]. Hence, it is plausible that allelic differences in genes involved in these mechanisms may affect mitochondrial function. *D. melanogaster* is also particularly amenable for studying the nuclear-encoded genomic control of naturally occurring variation in mitochondrial function since previous population studies have shown that the level of naturally occurring variation in the mtDNA of *D. melanogaster* is low compared to other Drosophila species [33,34]. Western hemisphere populations of *D. melanogaster* have been reported to be the least diverse with a single dominant haplogroup [34], suggesting that nuclear-encoded genes might explain some of the variation for mitochondrial respiration traits among the DGRP lines.

The 40 DGRP lines were previously quantified for transcript abundance [35] and their nuclear genomes have been sequenced [26]. This provided us with the opportunity to perform genome-wide association (GWA) and quantitative trait transcript (QTT) mappings to identify nuclear-encoded genes and molecular networks responsible for the control of naturally occurring variation in mitochondrial respiration. Using these approaches, we identified *sls* as a transcription regulator of mitochondrial respiration in *D. melanogaster*. The product of *sls* is a protein with homology to the NH2-terminal half of vertebrate titin [36]. As in mammals, Drosophila titin is a component of the muscle sarcomere and is required for both muscle and chromosome structure and elasticity [36]. Our results thus implicate a structural protein as a novel factor contributing to variation in individual mitochondrial respiration.

## Results and discussion

### Natural variation in mitochondrial respiration and efficiency among *D. melanogaster* lines

**Figure 1:**
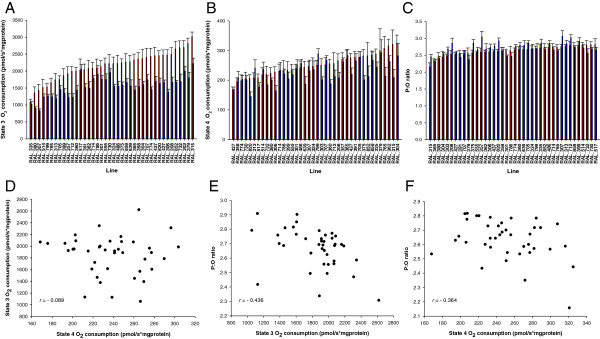
**Variation in mitochondrial respiration traits among 40 of the DGRP wild-derived inbred lines.** (Panels **A**-**C**) Distributions of line means for mitochondrial state 3 (Panel **A**) and state 4 (Panel **B**) respiration rates and P:O ratio (Panel **C**). Data represent means ± standard errors for *n* = 7 independent replicates. The red and blue bars depict females and males, respectively. (Panel **D**) Phenotypic correlation (*r*) between state 3 and state 4 respiration rates in the sex-pooled analysis. (Panel **E**) Phenotypic correlation between P:O ratio and state 3 in the sex-pooled analysis. (Panel **F**) Phenotypic correlation between P:O ratio and state 4 in females.

We found significant differences in the function of thoracic mitochondria isolated from the 40 DGRP lines (Figure 1 and Table 1). Our results indicate that 20%, 15%, and 17% of the variability in mitochondrial state 3, state 4, and P:O ratio, respectively, is attributed to genetic factors. We also observed significant differences between males and females, with females on average having higher mitochondrial respiration rates (Figure 1A and B) and coupling efficiency (Figure 1C) than males. Despite marked sexual dimorphism, the direction of the sex differences for state 3 and state 4 respiration rates was not affected by the genotype in our sample, as indicated by the absence of significant line-by-sex interactions (Table 1). A marginal effect, however, was found for P:O ratio (Table 1), suggesting that some of the loci regulating mitochondrial efficiency might have a sex-specific effect.

**Table 1:** Analysis of Variance of the mitochondrial traits for the 40 DGRP core lines

**Trait**	***Source***	***df***^***a***^	***MS***^***b***^	***F***	***P-value***	***σ***^***2 c***^
**State 3 respiration rate**	Line	39	1544364.61	6.18	<.0001	91195.80
	Sex	1	52476638.76	210.31	<.0001	Fixed
	Line × Sex	39	250041.46	1.25	0.1482	6979.40
	Error	495	200057.3			200057.30
**State 4 respiration rate**	Line	39	14874.15	3.29	0.0002	732.72
	Sex	1	88804.24	19.64	<.0001	Fixed
	Line × Sex	39	4524.33	1.05	0.3956	28.58
	Error	495	2138211.59			4319.60
**P:O ratio**	Line	39	0.24	2.62	0.0017	0.01
	Sex	1	0.58	6.31	0.0161	Fixed
	Line × Sex	39	0.09	1.41	0.0567	0.004
	Error	465	0.07			0.07

^*a*^Degrees of freedom. ^*b*^Mean Squares computed from Type III Sums of Squares. ^*c*^Restricted maximum likelihood estimates of variance component.

The sex-related differences in mitochondrial function observed in our study are consistent with the well-recognized differences between males and females in the control of substrate metabolism and energy homeostasis that occur across different species [37]. Also consistent with previous work is our finding that females have greater mitochondrial function than males. For example, Ballard et al. [38] showed higher mitochondrial efficiency in female *D. simulans* flies under similar mitochondrial respiration conditions used in our study. Furthermore, studies in mammalian models reported that female rodents had higher mitochondrial oxidative capacity and efficiency for substrate oxidation across several tissues [39-43]. The mechanisms underlying the sexual dimorphism in mitochondrial bioenergetic traits are not known, but the way evolution selects and optimizes certain genes for each sex has been indicated as a potential explanation [44]. Throughout evolution, genes from the mitochondrial genome and the *X* chromosome spend relatively more time under selection in females due to their asymmetric inheritance [44-46] and are therefore expected to be better optimized to function in females than in males [44]. Since females usually engage in more energetically demanding behaviors than males to attain reproductive success, it has been proposed that sexual differences may have arisen as an evolutionary adaptation to such differences in energetic demands [47].

Variation in mitochondrial respiration can be influenced by differences in mitochondrial density. To address this issue, we measured the activity of the marker enzyme citrate synthase (CS) in a panel of eight of the 40 inbred wild-type lines. The eight lines were selected to represent the range of variability in mitochondrial respiration rates and coupling efficiency seen in our sample. We measured CS activity in whole-fly homogenates and isolated mitochondria and calculated the mitochondrial protein density from the ratio between the CS activity of the whole-fly homogenates and that of the isolated mitochondria as described in [48]. Only one line was significantly different from the others in mitochondrial density (see Additional file 1). Also, there was no correlation between mitochondrial density and respiration rates or P:O ratio (Additional file 1), indicating that the variability in mitochondrial function among the DGRP lines is likely independent of the number of mitochondria.

### Phenotypic correlations between energy metabolism and life-history traits

Next, we asked whether there was a phenotypic correlation between mitochondrial respiratory rates and coupling efficiency among the 40 lines. We did not observe any correlation between state 3 and state 4 in both the sex-pooled analysis (*P* = 0.584) (Figure 1D) and the analysis stratified by sex (females: *P* = 0.716; males: *P* = 0.349). Given that in our study phenotypic correlations are mainly estimates of their genetic component, the results suggest that different genes regulate adaptive variation in these two traits. However, the P:O ratio was negatively correlated with state 3 in the sex-pooled analysis (*P* = 0.005) (Figure 1E) and with state 4 in females (*P* = 0.021) (Figure 1F). These latter findings are consistent with the previously reported dependence of mitochondrial efficiency on the rate of respiration in rat skeletal muscle [49] and the negative effect of the heat-producing proton leak on ATP production [6], and raise the important question of why inter-individual variability in mitochondrial coupling efficiency is maintained in natural populations. The 40 DGRP lines were previously assessed for several behavioral and life-history traits, including body weight [50], competitive fitness, copulation latency, locomotion, chill-coma recovery, starvation resistance, and lifespan [35], as well as energy storage and metabolic rate [50]. Hence, we tested for phenotypic correlations between these traits and the mitochondrial phenotypes. The results of the analysis are reported in Table 2. At *P* < 0.05, we observed a positive correlation between mitochondrial state 4 and starvation resistance (*P* = 0.005) in the analysis averaged across sexes and in males (*P* = 0.01). State 4 was also negatively correlated with copulation latency in females (*P* = 0.017). Furthermore, we found that state 3 was positively associated with glycogen levels in females (*P* = 0.03) and P:O ratio with body weight in males (*P* = 0.009). Although the correlations were not significant after Bonferroni correction for multiple tests (significance threshold: *P* < 0.003), they are in agreement with the physiological relationship among mitochondrial efficiency, nutrient availability, cell proliferation and death, and ROS production reported in mammals [51], and highlight the pivotal role of mitochondrial function in organismal energy homeostasis. Factors that adversely impact mitochondrial coupling and efficiency in eukaryotes have a deleterious impact on energy conservation [52]. Over 90% of cellular ROS are produced in the mitochondria [53]. The magnitude of ROS production is largely dependent on the proton-motive force across the inner membrane (membrane potential, or ΔΨ), so it can be strongly decreased by proton leak-induced mild uncoupling of mitochondrial OxPhos [54]. A balance must exist between generation and use of the electro-chemical gradient (ATP production and leak) so that the proton-motive force being generated under low energy requirements produces physiological levels of ROS involved in signaling and is still able to produce sufficient quantities of ATP. Otherwise the balance of cellular bioenergetics is lost. Excess ROS production under low energy requirement conditions of saturating reducing equivalents, high proton-motive force, well-coupled system, and exhaustion of antioxidant capacity, can oxidize mitochondrial components and impair mitochondrial function, which in turn, trigger apoptosis [51]. Additionally, ROS can negatively affect insulin signaling and therefore influence glycogen content [55,56], which we previously reported to be positively correlated with starvation resistance in the 40 DGRP line [50]. But in that same system greater efficiency would exist to produce ATP under conditions of high energy demand, such as growth, reproduction, muscle flight maintenance, and survival by starvation. 

**Table 2:** Phenotypic correlations between energy metabolism and life-history traits averaged across sexes (A), for females (B), and for males (C)

	**BW**^**a**^	**GLY**	**TAG**	**GLYC**	**MR**	**LC**	**FT**	**CL**	**SR**	**CC**	**LS**
**A**											
ST3	−0.210	0.267	−0.280	0.089	−0.149	−0.046	0.209	−0.089	0.017	−0.193	0.178
ST4	0.176	−0.041	−0.043	−0.210	0.073	0.012	−0.134	−0.271	0.439**	−0.097	0.146
P:O ratio	0.278	0.136	0.180	−0.010	0.203	0.161	−0.092	−0.260	0.059	0.254	−0.105
**B**											
ST3	−0.201	0.344*	−0.252	0.027	−0.178	−0.039	0.249	−0.214	−0.006	−0.293	0.157
ST4	0.122	−0.017	−0.236	−0.253	−0.052	−0.046	−0.099	−0.376*	0.204	−0.090	0.058
P:O ratio	0.074	0.039	0.284	0.016	0.099	0.120	−0.023	0.157	0.016	0.280	−0.086
**C**											
ST3	−0.177	0.090	−0.186	0.156	−0.077	−0.062	0.124	−0.111	0.079	−0.080	0.101
ST4	0.166	−0.034	0.184	−0.094	0.015	0.077	−0.136	−0.098	0.393**	0.287	0.147
P:O ratio	0.407**	0.217	0.011	−0.030	0.234	0.171	−0.117	0.056	0.151	0.157	0.004

^a^Pearson correlation coefficients. ST3, mitochondrial State 3 respiration; ST4, mitochondrial State 4 respiration; BW, body weight; GLY, glycogen; TAG, triacylglycerol; GLYC, glycerol; MR, metabolic rate; LC, Locomotion; FT, competitive fitness; CL, copulation latency; SR, starvation resistance; CC, chill-coma recovery; LS, lifespan. ^*^P ≤0.05; ^**^P ≤0.01.

### GWA analysis of mitochondrial respiration and efficiency traits

**Figure 2:**
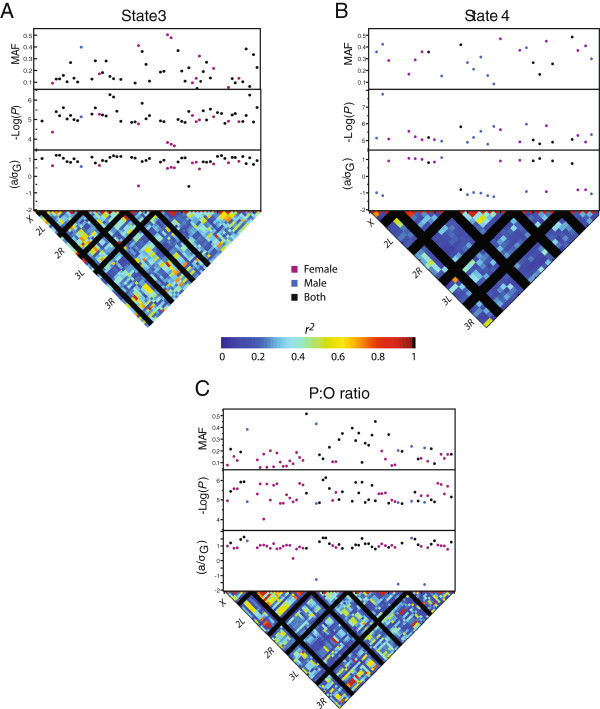
**GWA results for mitochondrial respiration and efficiency.** Significant SNPs (*P* ≤ 1×10^-5^) are plotted. The top panel shows the minor allele frequency (MAF) for each significant SNP. *P*-values are plotted as -Log_10_ (*P*) in the middle panel. Effect size normalized to genotypic standard deviation is plotted in the bottom panel. The lower triangle shows the distribution of linkage disequilibrium among SNPs as *r*^*2*^. Solid black lines identify the five major chromosome arms. (Panel **A**) State 3 respiration rates. (Panel **B**) State 4 respiration rates. (Panel **C**) P:O ratio.

For the GWA analyses, we used 1,312,183 SNPs with a minor allele frequency of at least 10%. The analysis averaged across sexes detected 40, 6, and 25 SNPs associated with variation in state 3, state 4, and P:O ratio, respectively, at a *P* ≤1 × 10^-5^ (Figure 2 and Additional file 2A). Given 1,312,183 SNPs, only 13 significant associations would be expected by chance at a *P* ≤1 × 10^-5^, thus the number of SNPs associated with state 3 and P:O ratio exceeded that expected by chance. We used gene-centered regression models to rank these SNPs in order of relative importance and to estimate the amount of phenotypic variance explained. We found that 2–3 SNPs could explain 31.1% of the phenotypic variance in state 3, 12.9% of the phenotypic variance in state 4, and 18.0% of the variance in P:O ratio (Table 3). Of the SNPs associated with state 3, state 4, and P:O ratio, 22, 2, and 25, respectively, were located within the transcription unit of annotated *D. melanogaster* genes (Additional file 2A). Because some genes were represented by two different SNPs and some SNPs were located in more than one gene, these analyses depicted 20, 2, and 27 nuclear-encoded candidate genes associated with variation in state 3, state 4, and P:O ratio, respectively. We also tested for association between SNPs and each trait using the data stratified by sex. Three, 12, and 18 additional genes regulating mitochondrial state 3, state 4, and P:O ratio, respectively, were identified by the sex-stratified association analyses at a *P* ≤1 × 10^-5^ (Additional file 2B). Only two genes, *capricious* and *Phosphodiesterase 1c*, were associated with SNPs affecting both mitochondrial OxPhos capacity and coupling efficiency. C*apricious* encodes a leucine-rich repeat protein that has been previously shown to play a role in the development of the tracheal tubes, the oxygen delivery system in insects [57]. Consistently, we detected five other genes, *O/E-associated zinc finger protein*, *center divider*, *headcase*, *karst*, and *Cad96Cb*, that are involved in tracheal development [58], suggesting that delivery of molecular oxygen has a major influence in ensuring efficient mitochondrial function and sufficient adaptation of the *D. melanogaster* muscle to different energy demand. *Phosphodiesterase 1c* encodes a calcium/calmodulin regulated protein with 3'5'-cyclic nucleotide phosphodiesterase activity that regulates intracellular levels of the cyclic nucleotide second messengers, cAMP and cGMP [59]. Although the mechanisms underlying the statistical associations are not yet known, this latter finding is particularly intriguing given the growing body of evidence from studies in mammals (reviewed in [32,60]) and yeast [61] suggesting a critical role for second messenger-mediated signal transduction mechanisms in the regulation of mitochondrial OxPhos. One example is represented by the cAMP/cAMP-dependent protein kinase (PKA) signal transduction pathway. Previous studies reported that cAMP not only activates the cytosolic PKA signaling, but also the mitochondrial signaling complex PKA/A-kinase-anchoring protein 1, which, in turn, regulates ATP production by phosphorylation of mitochondrial proteins, including subunits of complexes I and IV [62]. The regulation of complex IV by this signaling pathway is also mediated through its activation of transcription factors that control the expression of cytochrome *c* oxidase [32,61]. Similarly to PKA, the calcium-calcineurin phoshatase and protein kinase C signaling pathways have been implicated in the regulation of the OxPhos system in mammals [32]. Our analysis corroborates these previous findings showing that variation in *Calcineurin A1* and *CG31140* (predicted to encode a protein with diacylglycerol kinase activity) is associated with variation in state 3 and P:O ratio, respectively. 

**Table 3:** Multiple regression predictive models (A) and analyses of variance of haplotypes (B)

**Trait**	**Analysis**	**Variable**	**SNP Location**		**Estimate**	***t***	***P*-value**
		Intercept	*--*		2009.79	55.49	<0.0001
ST3	Sex Average	*3L*_651118	*Gale* (u3)		−290.43	−5.56	<0.0001
	*r*^2^ = 0.666	*3R*_18209173	*CG31169* (cds)		209.42	−5.78	<0.0001
		Intercept	*--*		237.31	44.29	<0.0001
ST4	Sex Average	*3R*_16981874	*SNF4Agamma* (in)		−15.71	−5.08	<0.0001
	*r*^2^ = 0.686	*2R*_3529122	*CG43340* (#)		18.15	5.84	<0.0001
		Intercept	--		2.693	124.52	<0.0001
P:O ratio	Sex Average	*2L*_18554474	*Pde11*(in)		0.046	3.71	0.0007
	*r*^2^ = 0.744	*3L*_2682962	*CG12187*(in)		−0.070	−5.12	<0.0001
		*3L*_9640097	*fry*(in)		−0.053	−3.90	0.0004
**B**							
**Trait**	**Analysis**	**Source of Variation**	***df***^***a***^	***MS***^***b***^	***F***	***P*-value**	***σ***^***2 c***^
		Haplotype	3	13648093	24.3	<0.0001	144977
ST3	Sex Average	Line (Haplotype)	36	577585	1.9	0.0014	19080
		Error	535	302190	--	--	302038
		Haplotype	3	127247	22.4	<0.0001	677.3
ST4	Sex Average	Line (Haplotype)	36	5689	1.3	0.1430	84.7
		Error	535	4498	--	--	4495.6
		Haplotype	6	1.26	21.8	<0.0001	15.0
P:O ratio*	Sex Average	Line (Haplotype)	33	0.06	0.9	0.6922	0.0
		Error	505	0.07	--	--	67.0

Markers are listed in the order in which they are entered in the model. Estimates of effects are for (Minor allele – Major allele). ST3, mitochondrial State 3 respiration; ST4, mitochondrial State 4 respiration. In, intronic; cds, coding sequence; #, missense; u3, 3’ UTR. ^*a*^Degrees of freedom. ^*b*^Mean Squares computed from Type III Sums of Squares. ^*c*^Restricted maximum likelihood estimates of variance component . * *σ*^2^ multiplied by 10^3^.

Intracellular cAMP production and signaling are regulated by G-protein coupled receptors (GPCRs), which represent the largest group of integral membrane proteins involved in signal transduction and exert a wide variety of biological functions, including neurotransmission, photoreception, chemoreception, metabolism, and cell differentiation and migration [63,64]. GPCRs predominantly exert their effects through interaction of their intracellular domains with heterotrimeric G**-**proteins [65]. Additionally, evidence has emerged in recent years indicating that GPCR signaling involves a complex network of interacting targeting and regulatory proteins that leads to cross-communication between separate signaling units [66]. For example, many GPCRs engage in crosstalk with receptor tyrosine kinases [67], the most relevant of which is the transactivation of the epidermal growth factor (EGF) receptor that allows GPCRs to initiate the Ras/Raf/MEK/ERK signaling pathway controlling cell proliferation, differentiation, and survival [67]. Interestingly, we found that the majority of the genes associated with state 3 and P:O ratio encode proteins involved in these signaling pathways. These include *dopamine receptor 2*, *tyramine β hydroxylase* (which encodes an enzyme catalyzing the last step in the synthesis of the invertebrate neurotransmitter octopamine [58]), *locomotion defects* (which encodes a protein that physically binds to the Gα subunit of the heterotrimeric G**-**proteins [58]), *star* (which encodes a transmembrane protein that is a member of the EGFR signaling [58]), *alphabet* (which encodes a serine/threonine phosphatase that acts as a negative regulator of the Ras/ERK pathway [58]), *still life* (which encodes a protein with Rho guanyl-nucleotide exchange factor activity [58]), and *RhoGEF* (see Additional file 2).

Another intriguing finding is the association between an intronic variant in the *SNF4/AMP-activated protein kinase gamma subunit* and variation in mitochondrial state 4 respiration rates (Additional file 2A). As mentioned above, state 4 in isolated mitochondria is strongly influenced by proton leak. AMP-activated protein kinase (AMPK) is a central sensor of cellular energy status and allocation [68]. As in mammals, Drosophila AMPK is an heterotrimer, with an alpha catalytic, a gamma regulatory, and a beta scaffolding subunits [69]. Drosophila AMPK is also activated by AMP and shares many of the same targets with mammalian AMPK [69]. Interestingly, recent work showed that flies deficient in AMPK are sensitive to starvation, a trait we have shown to be correlated to mitochondrial state 4 [68]. Previous *in vitro* studies also reported that loss of mitochondrial function and reduced ATP (highly correlated to mitochondrial proton leak) causes AMPK activation in flies [70]. Furthermore, AMPK increases the expression of uncoupling proteins in mammals [71,72]. Overall these observations suggest that population variation at the *SNF4/AMP-activated protein kinase gamma subunit* locus may be maintained as part of a mechanism controlling the production of ROS and therefore their signaling activities [73]. Additional data, however, will be needed to confirm this hypothesis.

Together, the above observations indicate that there is great utility in using the DGRP as a primary screen for follow-up testing of mutants of focal genes. Also, a notable result of our GWA study is that none of the nuclear genes encoding the subunits of the ETC enzyme complexes or the components required for the assembly and function of the respiratory chain was depicted by the SNPs that showed statistically significant association with the mitochondrial phenotypes. A growing body of evidence indicates that epistatic interactions between natural genetic variants in the mitochondrial and nuclear genomes affect mitochondrial function *per se*, as well as fitness and several life-history traits within and across populations [27,29,74-77]. Although these previous studies have made a significant contribution towards our understanding of the adaptive evolution of these complex traits, little is still known about the molecular nature of the nuclear-mitochondrial gene combinations. To this end, our results provide a new direction for future research seeking to identify the set of genetic variants involved in these mitonuclear epistatic interactions.

### Variation in *sls* has a mtDNA-independent effect on mitochondrial respiration

**Figure 3:**
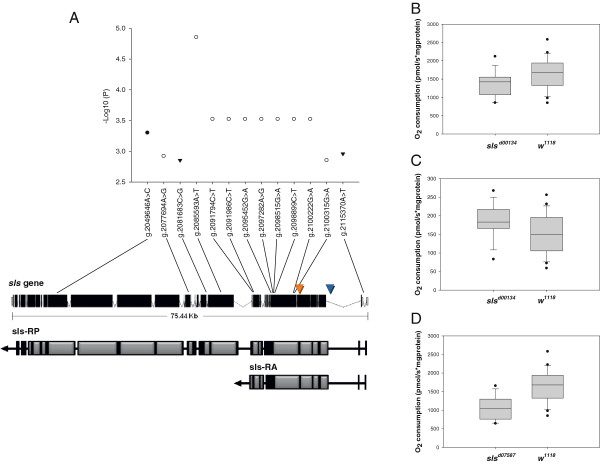
**Genetic variation in the*sls*gene associates with mitochondrial function.** (Panel **A**) The top panel shows the *sls* SNPs (*x*-axis) associated with mitochondrial state 3 (empty circle), state 4 (filled circle), and P:O ratio (triangle) at a nominal *P*-value (−Log_10_ (*P*), *y*-axis) of ≤ 1 × 10^-4^. The bottom panel reports a schematic representation of the *sls* gene on the third chromosome at cytological position 62C2-62C4 (NCBI Accession number AE014296.4) and of two alternative *sls* transcripts (sls-RP and sls-RA). The location of the *p[XP]* insertion sites that create the *sls*^*d00134*^ and *sls*^*d07587*^ mutations are indicated with an orange and blue arrowhead, respectively. (Panels **B**-**D**) In the analysis averaged across sexes, homozygous *sls*^*d00134*^ flies had lower state 3 (F_1,38_ = 5.37; *P* = 0.026) (Panel **B**) and higher state 4 (F_1,39_ = 4.32; *P* = 0.045) (Panel **C**) respiration rates than controls. Homozygous *sls*^*d07587*^ flies had lower state 3 respiration rates than controls (F_1,35_ = 20,61; *P* < 0.0001) (Panel **D**). In all panels, error bars represent standard errors for *n* = 14–24 independent replicates. Statistical significance was determined by a two-way ANOVA.

Our GWA analysis pinpointed *sls* as a novel candidate gene influencing inter-individual variability in state 3 respiration rate (Additional file 2A). *sls* encodes a protein with homology to the NH2-terminal half of vertebrate titin that is a component of the muscle sarcomere and is required for both muscle and chromosome structure and elasticity [36]. The 75.44 Kb *sls* gene consists of 37 exons (Figure 3A) and several alternative splicing have been described [78]. At least six Sls splice protein isoforms are expressed in the thoracic muscles of *D. melanogaster*, including the most abundant short isoform kettin (or sls-RA) (Figure 3A), which is present in the indirect flight muscles and contribute to their high passive stiffness necessary to generate stretch activation [78]. A total of 1693 SNPs in the *sls* gene have been previously reported to segregate in the 40 lines [26]*.* Of these, ten (synonymous coding), one (synonymous coding), and two (intronic and splice site intronic) were independently associated with state 3, state 4, and P:O ratio, respectively, at a nominal *P* ≤1 × 10^-4^ (which is a less stringent *P*-value threshold than the *P* ≤1 × 10^-5^ used in the GWA analysis) (Figure 3A). To confirm the direct effect of *sls* on mitochondrial function, we used two viable alleles of the gene, *sls*^*d00134*^ and *sls*^*d07587*^*,* which were established by the Exelixis Project via *P-*element (*XP*) insertion into the exon 6 and intron 2, respectively, of the *sls* gene in the *w*^*1118*^ strain [79]. We assessed mitochondrial function in mitochondria isolated from the thoraces of flies homozygous for each mutant allele and those homozygous for the corresponding wild-type allele. We found that homozygous *sls*^*d00134*^ flies, on average, had 17% lower mitochondrial state 3 (Figure 3B) and 18% higher state 4 respiration rates than controls (Figure 3C). Compared to controls, homozygous *sls*^*d07587*^ flies also displayed a 36% reduction in state 3 respiration rates (Figure 3C). No statistically significant differences were detected in the P:O ratio between any of the mutants and their controls as well as in the mitochondrial state 4 respiration rates between *sls*^*d07587*^ and control flies (data not shown). Additionally, we did not find any difference in mitochondrial protein density between *w*^*1118*^ and *sls*^*d00134*^ (*sls*^*d00134*^: 0.58 ± (standard error) 0.04 and *w*^*1118*^: 0.62 ± 0.06; *P* = 0.555) or *sls*^*d07587*^ (*sls*^*d07587*^: 0.33 ± 0.03 and *w*^*1118*^: 0.38 ± 0.05; *P* = 0.454) flies. Thus, these results are consistent with a regulation of mitochondrial respiration by *sls*. 

### Gene expression networks underlying variation in mitochondrial respiration

**Figure 4:**
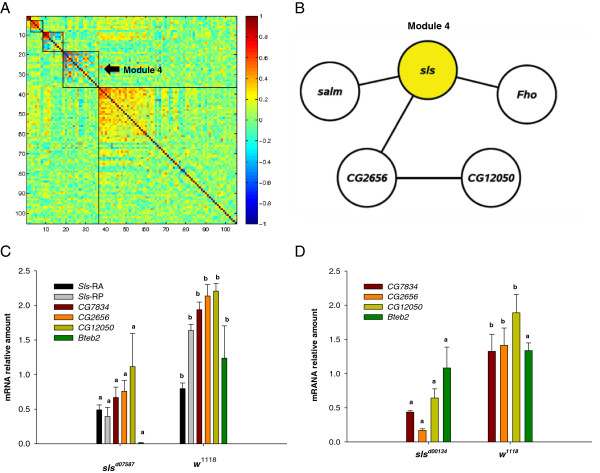
**Gene expression networks underlying variation in mitochondrial state 3 respiration rates.** (Panel **A**) Heat map of correlated probe sets after module formation for state 3 mitochondrial respiration rates (5 modules). Each point represents the correlation between two genes. The color scale bar indicates the values of the correlation. (Panel **B**) Network of correlated (|r|≥0.6) transcripts for state 3 module 4. Node showed as yellow in the network represents the *sls* gene*,* which was also identified by the GWA scan. (Panel **C** and **D**) Gene expression levels were measured by RT-qPCR using mRNA extracted from whole body of *w*^*1118*^ female flies and *sls*^*d07587*^ (Panel **C**) or *sls*^*d00134*^ (Panel **D**) female flies. Transcript levels of each gene were normalized to *rp49*. Values represent average of 4–6 independent replicates. Error bars represent standard error. Means designated by the same letter are not statistically significantly different from one another.

Genetic and functional studies in humans and animal models have provided strong evidence of the existence of co-expression genetic networks that control energy metabolism and homeostasis [80]. This is particularly true for muscle mitochondrial function considering the efficiency and adaptive plasticity of skeletal muscle bioenergetics in response to environmental changes [81]. The whole-body nuclear transcriptome profile of the 40 DGRP lines was previously assessed [35] under similar experimental conditions to this study. This previous work identified a total of 10,096 genetically variable transcripts [35]. Thus, we performed a genome-wide screen to determine whether variation in any of these transcripts was correlated with variation in mitochondrial function [15]. Because of the small sample size, we used a lenient *P*-value of 0.01 as a threshold for our analysis and found 105, 59, and 89 QTTs associated with state 3, state 4, and P:O ratio, respectively, in the analysis averaged across sexes (see Additional file 3). Given that the transcriptome data is not thorax-specific and the gene expression and mitochondrial data were collected in two different laboratories at different times, caution is needed in interpreting these results. But despite these limitations, we sought to use a weighted gene co-expression network analysis [35] to provide insight into how variation in the state 3 QTTs could give rise to variation in the trait. We identified five modules of correlated transcripts, ranging from 2 to 69 probe sets (Figure 4A and Additional file 4). Visualization of module 4 (Figure 4B) illustrates that *sls* is one of the most highly connected genes. Variation in *sls* gene expression is most highly correlated with variation in expression of *spalt major* (*salm*), which encodes a DNA binding transcription factor [58]. *salm* plays a critical role in the development of Drosophila indirect flight muscles [82]. *salm* induces the fate of fibrillar muscles (contained specifically in the indirect flight muscles) during Drosophila metamorphosis by regulating the gene expression profile of components of the sarcomere, including *sls* and *CG7834*[82]. The latter is predicted to encode a Drosophila ortholog of the human *Electron-transfer-flavoprotein, beta polypeptide* gene [58] and its transcript abundance is correlated with *sls* and other QTTs in the co-regulated state 3 module 4 (Additional file 4). 

Based on our results from the correlation network analysis, we hypothesized that the *sls*^*d00134*^ and *sls*^*d07587*^ mutations perturbed the same underlying transcriptional network as the natural variants. To test this idea, we compared the transcript abundance of four of the module 4 QTTs (*CG12050*, *CG2656, CG7834*, *Bteb2*, and *CG14291*) in homozygous mutant and control flies. Given that *sls*^*d07587*^ is a mutant generated by the insertion of a *P*-element in the intron 2 of the *sls* gene (Figure 3A), we also examined the effects of this *P*-element insertion on two of the *sls* transcript isoform variants (*sls*-RA and *sls*-RP). We found that the expression of both *sls* isoforms was significantly reduced in the *sls*^*d07587*^ flies (Figure 4C). In addition, consistent with the positive correlation observed in our network analysis, we observed that *CG12050*, *CG2656,* and *CG7834* had significantly lower expression in both *sls*^*d07587*^ (Figure 4C) and *sls*^*d00134*^ flies (Figure 4D) compared to controls. The molecular function of *CG12050* and *CG2656* is unknown; however, as mentioned above, *CG7834* is predicted to encode a protein with mitochondrial electron transfer flavoprotein (ETF) activity. Across different species, ETFs accept electrons from the FADH_2_ produced in the first step of the fatty acid β-oxidation and transfer them to the mitochondrial membrane-bound ETF ubiquinone oxidoreductase complex [83]. Thus, our finding suggest that *sls* might control mitochondrial respiration rates by affecting the respiratory chain [84].

## Conclusions

The present study demonstrates that natural populations of *D. melanogaster* exhibit genetic variation in mitochondrial respiration and efficiency. The GWA data also suggest that natural variation regulating long-term adaptation of *D. melanogaster* flight muscle mitochondrial function occurs in nuclear-encoded genes involved in a network of interconnected signaling pathways induced by extracellular stimuli, such as neurotransmitters, to maintain cellular homeostasis. Finally, the integrative genomic approach used in our study allowed us to identify *sls,* with homology to the human *Titin* (*TTN*) gene*,* as a novel hub gene responsible for the regulation of mitochondrial respiration in muscle sarcomere and to provide evidence that *sls* might act via the ETF ubiquinone oxidoreductase complex.

## Methods

### Drosophila stocks

The 40 unrelated wild-derived *D. melanogaster* inbred lines used in this study are a subset of the sequenced DGRP lines, which were established from a sample of isofemale lines collected in the Raleigh Farmer’s Market (North Carolina) and inbred to near-homozygosity by 20 generations of full-sib mating [26]. The *sls*^*d00134*^ and *sls*^*d07587*^ stocks were obtained from the Harvard Exelixis Stock collection (https://drosophila.med.harvard.edu/) and the *w*^*1118*^ (stock no: 6326) co-isogenic control line from the Bloomington Stock Center (http://flystocks.bio.indiana.edu). Each stock was maintained at constant parental density for at least two generations to minimize environmental effects. To control for larval density, we allowed the parents of the experimental flies to mate for 3 hours to generate egg collections on apple juice/agar medium in laying plates. After 24 hours, groups of 100 first-instar larvae were picked from the surface of the medium and put into replicate vials. To minimize the influence of genetic variation in reproduction on energy metabolism, all the phenotypic assays were performed on virgin flies that were randomly collected from the replicate vials for each line under brief CO_2_ exposure. For mitochondrial function assays, seven replicate vials per line were used, with each vial containing 20 single-sexed individuals aged 3–5 days. All lines were tested over a 2-year period and the lines and replicate vials were assayed in random order. Flies were reared in vials containing 10 ml of standard cornmeal, agar, molasses, and yeast medium, at a constant temperature of 25°C, relative humidity, and 12hr/12hr light/dark cycle.

### Mitochondrial respiration rate assay

Mitochondria were isolated from the thorax of the flies as described previously [20], with minor modification. All mitochondrial isolation steps were performed on ice. Live flies were chilled briefly on ice and thoraces were separated from the heads and abdomens. Isolated thoraces were placed into 200μl of ice-cold isolation buffer [250 mM sucrose, 5 mM Tris–HCl, 2 mM EDTA, 1% (w/v) bovine serum albumin (BSA), pH 7.4 at 4°C; [20]) supplemented with protease inhibitors (leupeptin 1mg/ml, aprotinin 1mg/ml and pepstatin 1mg/ml) in a 1.5 ml Eppendorf tube. The samples were pounded gently 126 times over a 2 minute period, using a motorized micromortar. Mashed flies were filtered through a 5 micron nylon mesh, and the volume was raised to 400μl by washing the nylon membrane with additional isolation buffer. After a cycle of low-speed centrifugation followed by centrifugation of the filtered solution for 10 min at 3000 g at 4°C, the pellet was re-suspended in 100μl of isolation buffer. Protein concentrations in the mitochondrial fractions were determined using a Lowry assay.

Using freshly isolated mitochondria, mitochondrial respiration assays were performed using a polarographic oxygen sensor (Oroboros oxygraph, OROBOROS® INSTRUMENTS**,** Innsbruck, Austria) with 0.2 mg/ml of freshly isolated mitochondria incubated in respiration medium (120mM KCl, 5mM KH_2_PO_4_, 3mM Hepes, 1mM EDTA, 1mM MgCl_2_, and 0.2% BSA, pH 7.2; [85]). Oxygen consumption rates were measured at 25°C [86]. As implemented by [20], we measured state 3 and state 4 respiration rates using the NAD^+^-linked substrates pyruvate 5mM/proline 5mM to deliver electrons into mitochondrial complex I. NAD^+^-linked substrates were added to the chamber and allowed to equilibrate for 1 min, followed by the addition of ADP at a concentration of 400μM to elicit ADP-dependent state 3. This was followed by the determination of the state 4 respiration rate, once all the added ADP had been exhausted and a steady state is reached [87]. P:O ratio, e.g. the relationship between ATP synthesis and oxygen consumption, was calculated as the amount of ADP consumed per oxygen being reduced during state 3.

All assays were performed within three hours of mitochondrial isolation. Data was analyzed using the software *DatLab* Version 4.1.0.8.

#### Quantitative genetic analyses

All statistical analyses were performed using SAS version 9.1. We used a mixed model ANOVA to partition variation in each trait among the inbred lines according to the model, *y* = *Âµ* + *L* + *S* + *LxS* + *ε*, where *μ* is the overall mean, *L* and *S* are the main effects of Line (Random) and Sex (Fixed), *LxS* is the random effect of sex-by-line interaction, and *ε* is the within-vial error variance. Pearson phenotypic correlations among traits were calculated by SAS PROC CORR using pooled data and data stratified by sex.

### Genotype-phenotype associations

The line mean of each mitochondrial parameter was associated with all segregating sites in the DGRP present in four or more DGRP lines, and having sequence coverage levels greater than two and less than thirty [26]. We used the ANOVA model *y* = *Âµ* + *M* + *S* + *M* × *S* + *L*(*M*) + *ε* to evaluate each segregating site, where *M* is the effect of SNP (marker) genotype, *L* is line, and *S* is sex. Genotype-phenotype associations were also performed for males and females separately using the reduced model *y* = *Âµ* + *M* + *ε*. We calculated the standardized effect size (*a*/*σ*_*G*_) as one-half the difference between marker classes divided by the overall genotypic standard deviation [88]. We used the *r*^2^ measure to compute linkage disequilibrium among significant markers [89].

To estimate the amount of genetic variance explained by the SNPs, we applied multiple regression models using gene-centered forward selection. We chose SNPs for the model that were highly significant and not in strong LD with each other (i.e., *P* < 10^-8^ for *r*^2^ between SNPs). We imputed SNP genotypes for markers with missing data. We fitted SNPs to the model, beginning with the most significant marker, until the *r*^2^ for variance was maximal. We identified haplotypes among replicate line means and analyzed the data for sexes combined and sexes separate using the model *y* = *Âµ* + *H* + *L*(*H*) + *ε*, where *H* is haplotype and *L* is line. We estimated the phenotypic variance explained as *σ*_H_^2^/(*σ*_H_^2^ + *σ*_L_^2^ + *σ*_E_^2^), where *σ*_H_^2^ is the among-haplotype variance component, *σ*_L_^2^ is the among-line variance component, and *σ*_E_^2^ is the error component.

#### Transcript-phenotype associations and transcriptional networks

The gene expression analysis in the 40 DGRP lines has been described previously [35]. Associations between genetically variable transcripts with each mitochondrial respiration trait were assessed by regression analysis as previously described [35]. Briefly, regression models of the form *y* = *Âµ* + *S* + *T* + *S* × *T* + *ε*, where *S* is sex, *T* is the mitochondrial trait, and *ε* is the error term, were computed for each probe set.

The genetic correlations between all QTTs associated with each trait at *P* < 0.01 were computed after removing the correlation between these transcripts and the trait. This was achieved by fitting the model *y* = *μ* + *E* + *S* + *E* × *S* + *ε* (*Y* is the trait, *E* is the covariate median log_2_ expression level, *S* is the sex effect and *ε* the residual error) and extracting the residuals to compute pair-wise transcript correlations for module construction [35]. Modules of transcripts associated with each trait with coordinated patterns of expression across the 40 lines were then quantified as described previously [90].

#### Quantitative RT-PCR

We isolated total RNA using the TriPure RNA isolation kit (Roche). Isolated RNA was then used to make cDNA, using the First Strand Synthesis kit (Invitrogen). We performed RT-qPCR using a SYBR Green Master mix and 50 ng total of cDNA per reaction and run in a Stratagene Mx3000P® qPCR machine. The primers used for qRT-PCR are listed in Additional file 5. Statistical significance was determined by the two-tailed Student’s *t* test.

## Abbreviations

DGRP: Drosophila Genetic Reference Panel; GWA: Genome-Wide Association; QTT: Quantitative Trait Transcript; ATP: Adenosine Triphosphate; OxPhos: Oxidative Phosphorylation; ETC: Electron Transport Chain; ROS: Reactive Oxygen Species; NADH: Nicotinamide Adenine Dinucleotide; FADH_2_: Flavin Adenine Dinucleotide; ADP: Adenosine Diiphosphate; UCP: Uncoupling Protein; mtDNA: Mitochondrial DNA; CS: Citrate Synthase; PKA: CAMP-Dependent Protein Kinase; GPCR: G-Protein Coupled Receptors; EGF: Epidermal Growth Factor; AMPK: AMP-Activated Protein Kinase; ETF: Electron Transfer Flavoprotein.

## Competing interests

The authors declare that they have non- financial competing interests.

## Authors’ contributions

DRM, PJL, and MD conceived the study and participated in its design and coordination. PJL, SB, DRM, and JCS performed research. STH, TFCM and MD analyzed the data. MD wrote the first draft of the manuscript. All authors read, critically revised the manuscript, and approved the final manuscript.

## Supplementary Material

Additional file 1 **Variation in mitochondrial protein density among eight of the 40*D. melanogaster*DGRP wild-derived inbred lines.** This file includes the distribution of line means for mitochondrial protein density in eight of the 40 DGRP lines and scatter plots of correlations between mitochondrial traits. Click here for file

Additional file 2 **List of SNPs significantly correlated with mitochondrial respiration and coupling efficiency in the analyses averaged across sexes (Panel A) and stratified by sex (Panel B).** This file includes SNP position, FlyBase accession number and name of the genes tagged by the SNPs and near intergenic SNPs, *P-*value from the ANOVA of the difference in trait means between the two SNP classes, minor allele frequency of the SNPs. Click here for file

Additional file 3 **List of transcripts significantly correlated with mitochondrial respiration and coupling efficiency.** This file includes Affymetrix Probe identification number, FlyBase accession number and name of the gene transcript, *P-*value from the regression analyses, and gene ontology information. Click here for file

Additional file 4 **Modules of correlated transcripts associated with mitochondrial function.** This file shows the average correlation of a transcript with all other transcripts in its module (degree) and the average correlation of all transcripts in the module (average degree). (XLSX 31 kb) Click here for file

Additional file 5 **Nucleotide sequence of primers used for qRT-PCRs.** This file reports the gene symbol, direction of the primer, and nucleotide sequence. (DOCX 11 kb) Click here for file
