# Supplementary material for: Nuclear genomic control of naturally occurring variation in mitochondrial function in Drosophila melanogaster
Source: BMC Genomics. 2012 Nov 22;13:659. doi: 10.1186/1471-2164-13-659 (PMC3526424; doi:10.1186/1471-2164-13-659)
Supplement: Additional file 1 — Variation in mitochondrial protein density among eight of the 40D. melanogasterDGRP wild-derived inbred lines. This file includes the distribution of line means for mitochondrial protein density in eight of the 40 DGRP lines and scatter plots of correlations between mitochondrial traits. [file 1471-2164-13-659-S1.docx]

**Additional Figure**

**Additional file 1** - **Variation in mitochondrial protein density among eight of the 40 *D. melanogaster* DGRP wild-derived inbred lines.** (Panel A) Distribution of line means for mitochondrial protein density in females. Data represent means ± standard errors for *n* = 5-6 independent replicates. Lines with the same letter do not differ significantly in mitochondrial density based on Tukey’s test. (Panel B) Phenotypic correlation (*r*) between mitochondrial protein density and state 3 respiration rate (*P* = 0.794). (Panel C) Phenotypic correlation between mitochondrial protein density and state 4 respiration rate (*P* = 0.334). (Panel F) Phenotypic correlation between mitochondrial protein density and P:O ratio (*P* = 0.931) .

B

B

A

A

A

A

B

B

B

B

B

**Lines**

B

A

**Line**

C

D
