# Supplementary material for: Nuclear genomic control of naturally occurring variation in mitochondrial function in Drosophila melanogaster
Source: BMC Genomics. 2012 Nov 22;13:659. doi: 10.1186/1471-2164-13-659 (PMC3526424; doi:10.1186/1471-2164-13-659)
Supplement: Additional file 5 — Nucleotide sequence of primers used for qRT-PCRs. This file reports the gene symbol, direction of the primer, and nucleotide sequence. (DOCX 11 kb) [file 1471-2164-13-659-S5.docx]

**Additional file 5 -Nucleotide sequence of primers used for qRT-PCRs**

| **Gene symbol** | **Primer direction** | **Nucleotide Sequence** |
| --- | --- | --- |
| *sls-*RA | Forward | CAGTTGGTCATCTCGGAGGC |
|  | Reverse | ATTGGACGCTGGTGGCTTT |
| *sls-*RP | Forward | GGTTTGCCCGAGTCAGATGG |
|  | Reverse | CGCAGCCACGAGGAGGTATT |
| *Bte2* | Forward | ACTTCTTTGCGACGGGTGG |
|  | Reverse | CGGACATACGGTCAGGTCATTG |
| *CG2656* | Forward | CAACCCGCTGACGGACATT |
|  | Reverse | GTCTGCCCATCTTTAGGTGAGTGA |
| *CG7834* | Forward | AAAGCCGACCTGGTCATCCT |
|  | Reverse | CGGGGTCTTGGTCTTGATTGT |
| *CG12050* | Forward | GAAGATGAGTCCCTGCGAAATG |
|  | Reverse | TGCCAGTGAAAGAGCGTGTTG |
